# Exploring Cervical Cancer‐Associated Vaginal and Cervical Microbiota via 16S rRNA Sequencing

**DOI:** 10.1002/mbo3.70412

**Published:** 2026-09-21

**Authors:** Guojiao Wang, Xiaotong Liu, Zimo Wang, Huadong Tian, Yan Li, Yuexi Luo

**Affiliations:** ^1^ The Second Clinical Medical College of North Sichuan Medical College, Beijing Anzhen Nanchong Hospital of Capital Medical University & Nanchong Central Hospital of Obstetrics and Gynecology Nanchong Sichuan China

**Keywords:** 16S rRNA gene sequencing, cervical cancer patients, metabolism, prevotella

## Abstract

Although the etiology of cervical cancer has been partially established, its pathogenesis remains a key research focus. Specific studies on the genital tract microbiota of cervical cancer patients in Southern China are still scarce. To address this research gap, the present study collected genital tract microbial samples from 16 cervical cancer patients and 16 healthy women. Analysis of cervical cancer‐specific microbiota was conducted via 16S rRNA gene sequencing. The study included 32 women (16 cervical cancer patients and 16 healthy controls) aged 45–65 at enrollment. Total genome DNA from samples was extracted using the hexadecyltrimethylammonium bromide (CTAB) CTAB method. The vaginal and cervical microbiota composition was determined by sequencing barcoded 16S rDNA gene fragments (V3‐V4), and a comparative bioinformatics analysis of the microbiome was performed. The study revealed a homogeneous microbial composition dominated by Lactobacillus in healthy women, whereas cervical cancer patients exhibited increased diversity with reduced Lactobacillus and enriched Prevotella. No significant differences were observed between sampling sites within each group. Functional predictions linked the cancer‐associated microbiota to metabolic pathways and the AEROBACTIN‐SYN‐PWY pathway. In conclusion, our findings suggest that these specific key microbial taxa and their related metabolic pathways contribute to the pathogenesis of cervical cancer and may serve as promising targets for clinical treatment and intervention.

## Graphical abstract

This 16S sequencing study identified a dysbiotic cervical microbiota (increased diversity, reduced Lactobacillus, enriched Prevotella) specifically linked to high‐risk HPV infection. These microbial alterations, shaped by the HPV‐driven microenvironment, offer novel targets for biomarkers and mechanistic research in cervical carcinogenesis.

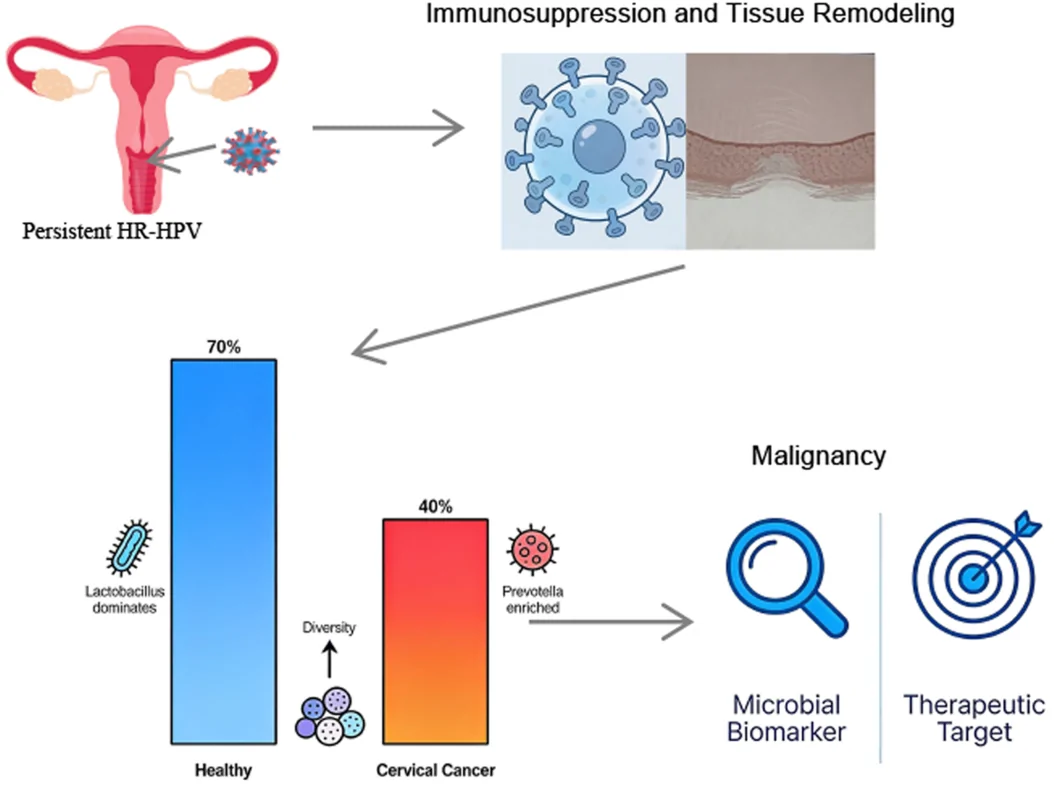

## 1 Introduction

Cervical cancer, ranked as the fourth most prevalent malignancy in women globally (Arbyn et al. 2020), poses a substantial challenge in gynecologic oncology research regarding its pathogenesis and prevention. According to recent statistics, over 600,000 new cases are diagnosed annually worldwide, with nearly 90% occurring in resource‐limited developing countries (Arbyn et al. 2020). In China, the incidence of cervical cancer is shifting toward younger age groups, significantly threatening women's health and quality of life (Cai et al. 2010). Although persistent infection with high‐risk human papillomavirus (HPV) is recognized as the primary etiological factor (Arbyn et al. 2020), only a small proportion of infected individuals progress to precancerous lesions or invasive carcinoma (Wang et al. 2024). These findings indicate that additional key factors beyond HPV contribute to cervical carcinogenesis. Recently, the interactions between microbiota and cancer have been increasingly explored. Accumulating evidence indicates specific microbial signatures in HPV persistence and cervical carcinogenesis (Ivleva and Grivennikov 2022), yet the potential link between microbial dysbiosis and cervical carcinogenesis requires further elucidation.

Current evidence confirms that the reproductive tract of healthy women is dominated by the genus Lactobacillus, which exerts protective effects through maintaining an acidic environment, competitively inhibiting pathogens, and modulating immune responses (Ravel et al. 2011). Notably, the vaginal microbiota of cervical cancer patients is marked by a notable reduction in Lactobacillus abundance alongside aberrant proliferation of anaerobic bacteria, including Gardnerella and Prevotella (Bai et al. 2024; Li et al. 2023; Liu et al. 2022). Such dysbiosis is closely associated with persistent HPV infection and the progression of cervical intraepithelial neoplasia (CIN) (Cui et al. 2025). Currently, existing studies face two major limitations. First, epidemiological studies have consistently identified bacterial vaginosis (BV) as a risk factor for cervical cancer, demonstrating that women with BV have a significantly higher risk of developing cervical neoplasia (Liang et al. 2019). The molecular mechanisms underlying the microbial functional gene expression and host–microbiota interactions mediated by microbial metabolites remain unclear. Second, conventional microbial culture techniques, constrained by low sensitivity and limited coverage, fail to comprehensively characterize the dynamic evolution of complex microbial ecosystems.

To address these mechanistic and methodological gaps, this study integrated high‐throughput 16S rRNA gene sequencing (Callahan et al. 2019) to systematically examine the compositional and functional differences in the vaginal‐cervical microbiota between cervical cancer patients and healthy controls in a southern Chinese population. Key microbial biomarkers associated with cancer progression were identified. By investigating associations between microbial structure‐function networks and host clinical phenotypes, this study aimed to elucidate the role of reproductive tract microbiota dysbiosis in driving cervical carcinogenesis through potential molecular pathways, identify microbial biomarkers with early‐risk predictive potential, and establish a mechanistic basis for precision interventions based on microecological remodeling.

## 2 Materials and Methods

### 2.1 Study Participants

A total of 65 women visiting the Gynecology Department of Nanchong Hospital, Beijing Anzhen Hospital, and Capital Medical University between January and June 2024 were initially screened for eligibility. A consecutive sampling strategy was adopted to minimize selection bias. All eligible women who visited the outpatient service during the study period were invited to participate in this study. Among them, 32 women were enrolled after applying the inclusion and exclusion criteria. All participants voluntarily agreed to participate and provided written informed consent. The study was approved by the Ethics Committees of the participating hospitals and was conducted in accordance with the principles of the Declaration of Helsinki. The sample size of 32 was determined because 32 participants consecutively recruited from the participating hospitals during the 6‐month study period (January to June 2024) were eligible. No formal a priori sample size calculation was performed, as this was an exploratory study. Based on vaginal discharge microscopy and cervical biopsy histopathology, the included individuals were stratified into two groups: cervical cancer patients (*n* = 16) and healthy controls (*n* = 16). Inclusion criteria were women aged 45–65 years who were non‐menstruating and not pregnant. Notably, HPV DNA testing and genotyping were performed on cervical exfoliated cell samples from all participants. Exclusion criteria included: (i) use of antibiotic or antimicrobial vaginal agents within 1 week, or vaginal douching within 3 days; (ii) presence of concurrent bacterial vaginosis, candidal vaginitis, or urinary tract infections; (iii) history of sexually transmitted infections (such as mycoplasma, chlamydia, gonorrhea, syphilis, genital herpes, and trichomoniasis); (iv) prior cervical lesions or hysterectomy; (v) systemic diseases (such as diabetes, autoimmune disorders, and malignancies); (vi) smoking or alcohol consumption.

### 2.2 Sample Collection and DNA Extraction

During gynecological examinations, three sterile swab samples were collected separately from the vaginal wall and cervical os and immediately stored at ‐80°C for DNA extraction. Total genomic DNA was extracted via the cetyltrimethylammonium bromide method. DNA concentration was measured by NanoDrop, and DNA quality was assessed via 1.2% (w/v) agarose gel electrophoresis. Qualified DNA samples were subsequently used for 16S rRNA gene sequencing.

### 2.3 16S rRNA Gene Sequencing

#### 2.3.1 Polymerase Chain Reaction (PCR) Amplification

The highly variable V3‐V4 region of the bacterial 16S rRNA gene (about 480 bp in length) was selected for sequencing. PCR amplification was performed using 16S rRNA V3–V4 region‐specific primers: 338 F (5′‐barcode+ACTCCTACGGGAGGCAGCA‐3′) and 806 R (5′‐GGACTACHVGGGTWTCTAAT‐3′) (Klindworth et al. 2013).

#### 2.3.2 Purification

PCR products were purified using magnetic beads. DNA binding: samples were mixed with magnetic beads to allow DNA binding. First wash: 200 μL of 80% ethanol was added, incubated briefly, and the supernatant was discarded. Second wash: the ethanol wash step was repeated, and the supernatant was completely removed. Dry: the beads were air‐dried at room temperature for 5 min, until cracks appeared. Elute: 25 μl Elution Buffer was added, and the beads were thoroughly resuspended. Recover: The tube was placed on a magnetic stand for 5 min, and the supernatant was transferred to a new tube.

#### 2.3.3 Fluorescence Quantification of PCR Products

The PCR amplification products were subjected to fluorescence quantification. The fluorescent reagent used was the Quant‐iT PicoGreen dsDNA Assay Kit, and the quantification was performed using a Microplate reader (BioTek, FLx800).

#### 2.3.4 Library Preparation

Sequencing libraries were constructed using the Illumina TruSeq Nano DNA LT Library Prep Kit.

#### 2.3.5 High‐Throughput Sequencing

Paired‐end sequencing (2×300 bp) was conducted on an Illumina MiSeq platform using the MiSeq Reagent Kit v3 (600‐cycle).

### 2.4 Bioinformatics Analysis

#### 2.4.1 Raw Data Processing

Quality control: Raw reads were subjected to quality assessment. Samples that failed quality control were excluded from downstream analysis. No samples were re‐sequenced or supplemented due to the limited availability of original samples. Demultiplexing: reads that passed the initial quality screening were demultiplexed into libraries and samples based on index and barcode sequences. Primer trimming: primer sequences were removed using qiime cutadapt trim‐paired (QIIME2 v2024.5) (Bolyen et al. 2019). Reads without primer matches were discarded. Denoising and merging: the DADA2 algorithm was implemented via qiime dada2 denoise‐paired to perform quality filtering (trunc‐len‐f = 240, trunc‐len‐r = 200, maxEE=2), error correction (denoising), paired‐end read merging, and chimera removal (Callahan et al. 2016). Amplicon sequence variant (ASV) table construction: feature sequences and frequency tables from all libraries were merged, and singleton ASVs (abundance ≤ 1) were removed. Sequence length analysis: the length distribution of high‐quality sequences was statistically summarized and visualized.

#### 2.4.2 Taxonomic Composition Analysis

Taxonomic annotation was performed using the Greengenes database (Release 13.8 (DeSantis et al. 2006) and the QIIME2 (v.2024.5) classify‐sklearn algorithm (Bokulich et al. 2018). Representative sequences of ASV and operational taxonomic unit (OTU) tables were annotated with a pre‐trained Naive Bayes classifier under default parameters in QIIME2. Notably, not all feature sequences were annotated to the genus level due to incomplete coverage of microbial diversity in the reference databases (unassigned labels), lack of precise taxonomic information in reference sequences (unidentified, uncultivated, uncultured, incertae sedis), and resolution limitations imposed by sequencing read length (unclassified at genus or species levels).

Phylogenetic trees were built via the QIIME phylogeny align‐to‐tree‐mafft‐fasttree pipeline. Multiple sequence alignment was performed by MAFFT (Katoh 2002), phylogenetically uninformative sites were masked, and maximum‐likelihood trees were generated with FastTree (Price et al. 2009). Phylogenetic analysis was not applied to internal transcribed spacer or functional gene sequences.

Rarefaction was conducted to normalize sequencing depth (Heck et al. 1975; Kemp and Aller 2004). ASV/OTU tables were subsampled to an equal sequencing depth. Relative abundances of ASVs/OTUs were calculated at this depth. Taxonomic composition was visualized using the qiime taxa barplot command with singleton‐filtered feature tables, generating stacked bar plots to provide an overview of sample‐ and group‐specific profiles.

#### 2.4.3 Analysis of Overall Sample Diversity

Microbial diversity at the ASV/OTU level was comprehensively assessed using both alpha and beta diversity indices to characterize diversity within habitats (alpha diversity) and between habitats (beta diversity), respectively.

Alpha‑diversity analysis was performed on the non‑rarefied ASV/OTU table using the “qiime diversity alpha‑rarefaction” command with the following parameters: ‑‑p‑steps 10, ‑‑p‑min‑depth 10, and ‑‑p‑iterations 10. The minimum rarefaction depth was set to 10 sequences, with the maximum depth (‑‑p‑max‑depth) automatically defined as 95% of the sequencing depth of the sample with the lowest read count in the dataset. Ten evenly spaced depth values were sampled between these bounds, and at each depth, rarefaction was repeated 10 times to compute alpha‑diversity indices. The mean values at the maximum rarefaction depth were taken as the final alpha‑diversity metrics. Subsequently, beta‑diversity analysis was conducted on the rarefied table using the “qiime diversity core‑metrics‑phylogenetic” command, which generated four distinct distance matrices. Principal coordinate analysis (PCoA) was then performed based on each matrix. All results were exported as QZV files. For visualization, the QZV files can be uploaded by dragging them into the designated area on the online platform https://view.qiime2.org/. Boxplots were generated using R software (version 4.3.1) [R Core Team, 2024], and the ggplot2 (version 3.4.2) and ggpubr (version 0.6.0) packages were used for data visualization and statistical annotation to visually compare alpha diversity differences among sample groups. Beta diversity indices were used to assess differences between habitats, reflecting dissimilarities among samples. Principal component analysis (PCA) was applied to visualize compositional differences in species abundance across sample groups. To further compare inter‐sample variations in species composition and display abundance distribution patterns, heatmaps were utilized for species composition analysis. Venn diagrams were constructed using the R package VennDiagram (version 1.7.3) to analyze community overlap and identify taxa unique to or shared among different sample groups, based on presence or absence data rather than abundance values. The number of exclusive and shared ASVs/OTUs among sample groups was calculated, and the flower plots were generated to simplify the visualization of multi‐group comparisons.

#### 2.4.4 Association Network Construction and Functional Prediction

Association network analysis was performed to identify key microbial taxa based on species distribution patterns across samples. It served as a well‐established approach for inferring microbe‐microbe relationships (Faust and Raes 2012). This methodology aimed to detect inherent co‐occurrence or co‐exclusion patterns within microbial communities driven by spatiotemporal dynamics or environmental processes. Using non‐rarefied ASV/OTU abundance data from all project samples, features with fewer than 10 total sequences or presence in fewer than five samples were excluded. Correlation matrices were generated via the SparCC algorithm (Friedman and Alm 2012), and statistically significant edges were determined based on random matrix theory thresholds. Networks were constructed with igraph (Csardi and Nepusz 2006). For each treatment group (Treat), subnetworks were derived using igraph's induced subgraph function based on ASV/OTU presence/absence. Negative correlations were removed to construct co‐occurrence networks, which underwent modular decomposition via igraph's multi‐level modularity optimization algorithm. Modularity indices were calculated, and networks were visualized using ggraph. Exportable.gml files were generated for further customization in Gephi (Bastian et al. 2009) and Cytoscape (Shannon et al. 2003). Dominant taxa subnetworks were constructed using the top 50 most abundant nodes (default).

Functional potential was predicted via PICRUSt2 (Phylogenetic Investigation of Communities by Reconstruction of Unobserved States) (Douglas et al. 2020), specifically targeting Kyoto Encyclopedia of Genes and Genomes (KEGG) Orthologs. 16S rRNA feature sequences were aligned to reference sequences to build a phylogenetic tree. The Castor hidden state prediction algorithm inferred gene family copy numbers for features by extrapolating from reference‐associated copy numbers within the phylogenetic framework. Sample‐specific gene family abundances were calculated by integrating inferred copy numbers with feature abundance profiles. Taxonomic annotations were retained in hierarchically structured outputs (without merging functional units across distinct feature sequences) to enable function‐taxon correspondence analysis. Finally, gene families were mapped to metabolic pathways using MinPath (default) to infer pathway presence and abundance.

### 2.5 Statistics

SPSS software (version 25.0) was used to analyze demographic and clinical data. The chi‐square test was applied for categorical variables, and Student's t‐test for continuous variables. The ANOSIM test was applied for intergroup comparison of the 16S rRNA gene. Statistical analyses were carried out using R software (v.4.3.3). A *p*‐value < 0.05 was considered statistically significant.

## 3 Results

### 3.1 Differences in Microbial Composition Between Cervical Cancer Patients and Healthy Controls

**Figure 1:**
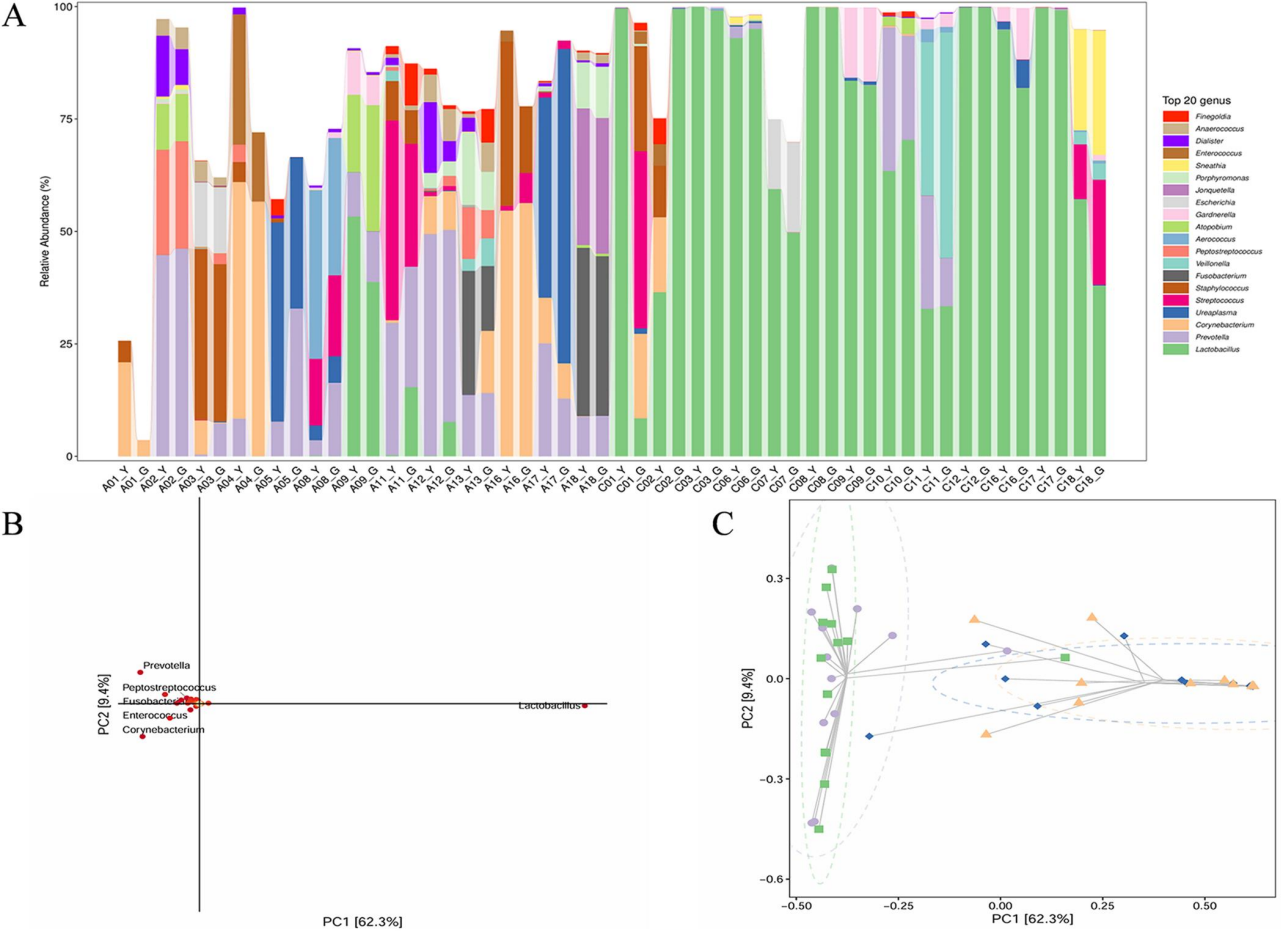
(A) Genus‐level taxonomic composition. Y‐axis: relative abundance (%) (0%–100%, proportion of each microbial genus per sample). X‐axis: top 20 genera grouped by cervical cancer (A‐Y: vaginal wall, A‐G: cervical os) and control cohorts (C‐Y: vaginal wall, C‐G: cervical os); (B) Species loading plot of PCA. Each point represents a genus. Coordinates indicate its contribution to inter‐sample variance along principal components dimensions (variance explained percentages in parentheses). Physical axis scales are proportional to explained variance ratios. Contribution magnitude correlates positively with cumulative distance from axes (color gradient: yellow→ red); (C) Sample ordination plot. Each point represents a sample (color‐coded by group). Physical axis scales are normalized (unit ratio = 1). Proximity between points reflects similarity in species abundance profiles along respective dimensions. Dashed ellipses denote 95% confidence regions for sample groups.

The female reproductive tract was dominated by Lactobacillus spp., which played a crucial role in maintaining microecological homeostasis. This study employed 16S rRNA high‐throughput sequencing to compare microbial composition differences between cervical cancer patients and healthy controls, aiming to identify carcinogenesis‐associated keystone genera and their metabolic features. The study initially included 16 females with cervical cancer (“Cancer”=A group) and 16 healthy females (“Control”=C group) (Table S1). Due to the poor quality of individual samples, three samples from each of the two groups were excluded, resulting in a final sample size of *N* = 13 per group in the main text. No significant difference in BMI, age, smoking, drinking, number of pregnancies, number of deliveries, number of abortions, age at menarche, menstrual cycle frequency, or menstrual duration between the two groups was observed (Table 1). Analysis of genus‐level composition from 16S rRNA sequencing (Figure 1 A) and Taxonomic Tree with Abundance Information (Figure S1) revealed that healthy controls were dominated by Lactobacillus, with no significant inter‐individual differences within the group. The healthy control group consistently exhibited Lactobacillus dominance, with minimal variation among individuals. In contrast, all cervical cancer cases were positive for high‐risk HPV, while the controls were universally HPV‐negative. This clear contrast in infection status allowed us to analyze the specific microbiome shifts attributable to HPV as the principal pathogenic driver. Our observations indicate that the progression from an HPV‐negative healthy state to an HPV‐positive cervical cancer state is accompanied by marked structural dysbiosis within the cervical microbial community. In contrast, cervical cancer patients exhibited a significant decrease in the relative abundance of *Lactobacillus* (median (IQR) (Cancer vs. Control, 12.5% [8.2%–18.3%] vs. 72.5% [58.3%–84.1%], *p* < 0.001)) and a significant increase in *Prevotella (*(28.3% [19.7%–35.6%] vs. 6.8% [3.2%–11.4%], *p* < 0.001)). The cancer group demonstrated higher microbial diversity than healthy controls. Inter‐individual microbiota variations were observed within the cancer group compared to controls. No substantial differences were detected between vaginal wall and cervical os microbiota within either healthy or cancer groups. *Lactobacillus* dominated the reproductive tract microbiota in healthy controls, with a median relative abundance of 72.5% (IQR: 58.3%–84.1%), whereas in cervical cancer patients, its abundance was significantly reduced to 12.5% (IQR:8.2%–18.3%, *p* < 0.001). Anaerobic genera, including Prevotella, Peptostreptococcus, Fusobacterium, Enterococcus, and Corynebacterium, were enriched in the cancer group (Figure 1B). Healthy individuals maintained stable microbiota across anatomical sites, while cervical cancer patients exhibited site‐specific heterogeneity (Figure 1C). This spatial variation reflected differential progression patterns of carcinogenesis and underscored the importance of maintaining acidic microenvironments within reproductive tract niches. To isolate the effect of the viral factor, based on the HPV genotyping results, the cervical cancer group (the Cancer group) was divided into the following two subgroups: ① HPV16+ group: all samples in which HPV16 was detected (A01, A02, A03, A04, A05, A08); ② HPV + /non‐16 group: all samples that tested positive for high‐risk HPV but were negative for HPV16 (A09, A11, A12, A13, A16, A17, A18). Analysis revealed that the microbial community structure remained comparable between these subtype groups, though underlying differences in viral load or clinical stage (HPV16+ gruop VS HPV + /non‐16 group) (Figure 1A ) were not significantly different from each other, but were significantly different from the HPV‐ group. This indicated that the impact of high‐risk HPV infection (regardless of subtype) on the cervical microecology may be greater than the differences among subtypes, and the common pathway of HPV carcinogenesis may be related to the disorder of the microbiota.

**Table 1:** Demographic and clinical characteristics of participants.

	Cancer	Control	*p*‐value
	*N* = 13	*N* = 13	
Age (years)	56.38 (5.33)	52.08 (3.12)	0.019^a^
BMI (kg/m^2^)^d^	24.78 (4.69)	23.22 (2.58)	0.305^a^
Smoke: N	13 (100%)	13 (100%)	‐b
Drink: N	13 (100%)	13 (100%)	‐b
Number of pregnancies	3.46 (1.71)	3.15 (1.14)	0.595^a^
Number of deliveries	2.38 (1.12)	1.46 (0.78)	0.022^a^
Number of abortions	1.08 (1.66)	1.69 (1.18)	0.286^a^
Age at menarche (years)	13.46 (1.05)	13.15 (1.07)	0.466^a^
Menstrual cycle frequency^c^ (days)	29.00 (2.45)	29.92 (2.25)	0.327^a^
Menstrual duration (days)	6.08 (1.11)	5.38 (0.77)	0.078^a^

Data are presented as Mean (SD) for continuous variables and n (%) for categorical variables.

^a^ *P*‐values were calculated using Student's t‐test for continuous variables and the chi‐square test was applied for categorical variables

^b^ Statistical testing was not applicable as the characteristic was invariant across groups (all participants reported no smoking/alcohol consumption).

^c^ “Frequency” refers to the average length of the menstrual cycle in days.

^d^ BMI: body mass index

### 3.2 Differential Intra‐ and Inter‐Group Microbial Characteristics

**Figure 2:**
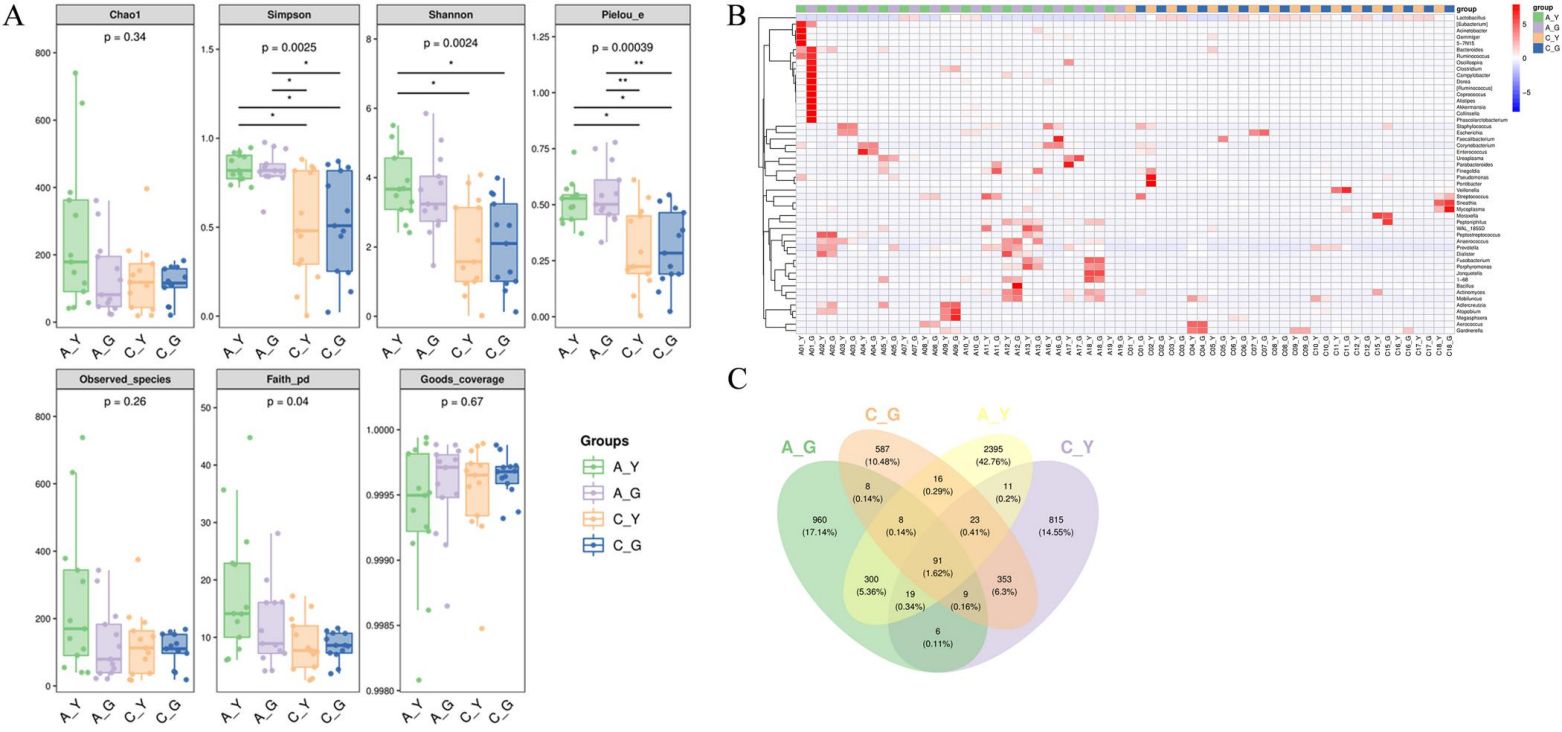
(A) Boxplots of alpha diversity indices. Each panel represents one index (labeled in gray header). X‐axis: group categories; Y‐axis: index value. P‐values from Kruskal‐Wallis tests are shown below the index labels. (B) Genus‐level abundance heatmap with clustering. Sample clustering: samples are ordered by UPGMA clustering based on Euclidean distance of compositional data (or by group/default order). Taxon clustering: genera are ordered by UPGMA clustering of Pearson correlation coefficients (or by mean abundance). Color gradient: red = higher relative abundance; blue = lower relative abundance. (C) Venn diagram of ASV/OTU distribution. Each ellipse represents a sample group. Overlapping regions indicate shared ASVs/OTUs between groups. Numbers denote ASV/OTU counts per region. The percentages represent the number and proportion of shared or unique taxonomic units (e.g., ASVs/OTUs) among different groups.

Inter‐individual microbial heterogeneity was further examined using appropriate statistical tests. Alpha‐ and beta‐diversity metrics were utilized to quantify within‐sample (α) and between‐sample (*β*) diversity, respectively, providing a comprehensive assessment of overall community diversity. Alpha‐diversity analyses revealed the following (Figure 2A ): (i) Chao1 and observed species indices did not differ between groups, indicating comparable species richness. Cervical cancer samples thus did not exhibit a net gain or loss in bacterial numbers but rather a shift in community composition. (ii) Pielou's evenness was significantly lower in group A (cervical cancer) than in group C (healthy controls), suggesting reduced dominance and a more even distribution in the cancer‐associated microbiota. (iii) Shannon and Simpson indices showed significantly elevated diversity in the cancer cohort. (iv) Faith's phylogenetic diversity (Faith'PD) was higher in group A, reflecting a more dispersed evolutionary lineage spectrum. This may reflect concomitant colonization by multiple opportunistic pathogens and a fragmented community structure following the collapse of the Lactobacillus‐dominated stable state. (v) Good's coverage values were equivalent across groups, confirming consistent sequencing depth and excluding technical bias as a source of the observed differences.

Heat‐map visualization (Figure 2B) demonstrated that Lactobacillus exhibited intense red signals (high abundance) in normal samples (C‐Y and C‐G), peaking in vaginal‐wall swabs (C‐Y), whereas it appeared blue (virtually absent) across cancer samples (A‐Y and A‐G). Conversely, obligate anaerobic pathogens, including Fusobacterium, Prevotella, and Peptostreptococcus, formed contiguous dark‐red patches in cervical‐os cancer samples (A‐G) and scattered red foci in vaginal‐wall cancer samples (A‐Y), indicating clustered enrichment in (A‐G) versus focal enrichment in (A‐Y).

To further compare the species composition differences between samples and investigate which species are shared or unique among different samples (Figure S2).

Venn‐diagram analyses were further applied to identify shared versus unique taxa among sites. Normal vaginal‐wall swabs (C‐Y) harbored the richest community, whereas cancer vaginal‐wall swabs (A‐Y) were the least diverse. Healthy vaginal‐wall (C‐Y) and cervical‐os (C‐G) samples shared a large core, underscoring high inter‐site similarity under homeostatic conditions. By contrast, cancer vaginal‐wall (A‐Y) and cervical‐os (A‐G) samples shared markedly fewer taxa, reflecting increased spatial heterogeneity during malignancy. Only a minimal core was common to all four groups, signifying a fundamental restructuring of the microbiota and the loss of numerous health‐associated core taxa. The negligible overlap between cancer and normal cohorts further indicated a profound microbial reconfiguration driven by cervical carcinogenesis (Figure 2C ).

To assess the overall structural differences in the cervicovaginal microbiota among groups, analysis of similarities (ANOSIM) was performed based on Bray–Curtis distances. The results revealed a significant but moderate separation between the cervical cancer group and the healthy control group (*R* = 0.218, *p* = 0.001, based on 999 permutations). The R‐value, which ranged from −1 to 1, indicated the degree of separation between groups. An R‐value close to 1 suggested complete separation (i.e., between‐group dissimilarity greater than within‐group dissimilarity), while an R‐value near 0 indicated no distinct separation. Here, the *R* = 0.218 indicated that although the groups were statistically distinguishable, the separation was relatively modest. These results suggested that while the overall microbial community structure differed between cancer patients and healthy controls, there was still considerable within‐group variability. The *p* = 0.001 confirmed that this observed separation was highly unlikely to have occurred by chance.

### 3.3 Functional Prediction of Key and Differential Taxa

**Figure 3:**
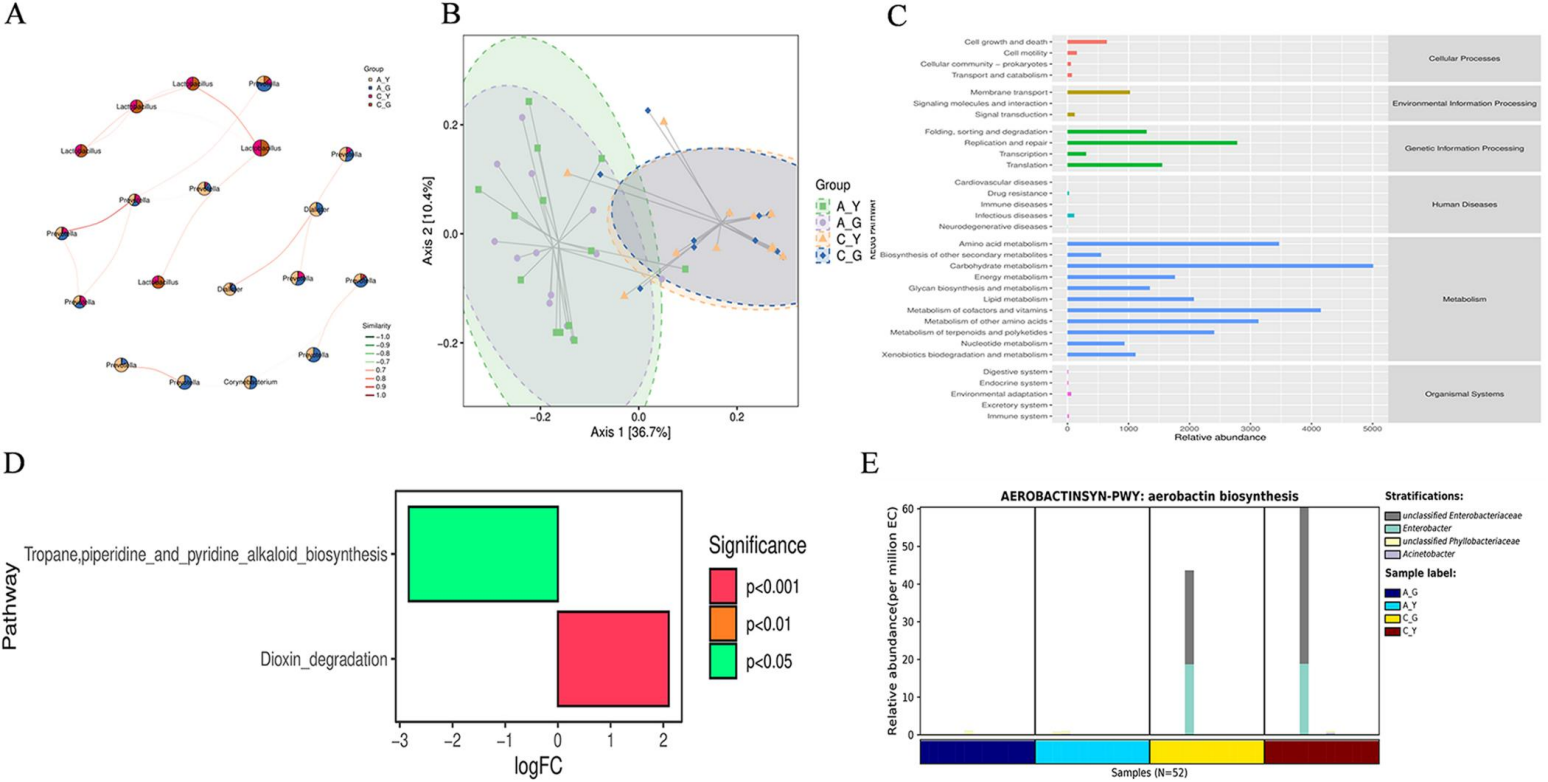
(A) Dominant taxon subnetwork with group abundance pie charts. Nodes represent ASVs/OTUs (top 50 by mean abundance). Node size is proportional to abundance, expressed as log_2_(CPM/n). Edges indicate correlations: red = positive, green = negative. (B) PCoA of functional units based on Bray‐Curtis distance. Each point represents a sample (color‐coded by group). Percentages in parentheses on the axes indicate the proportion of variance in the sample dissimilarity (distance matrix) explained by that axis. Axes are scaled uniformly (unit ratio = 1) for visualization. Distances should be interpreted as projections rather than direct Euclidean distances. Points closer together along an axis indicate more similar functional composition in that dimension. Dashed ellipses represent 95% confidence regions for sample groups. Key separation: Cancer (A‐Y/A‐G) versus healthy (C‐Y/C‐G) along Axis 1 (36.7% variance explained). (C) Predicted KEGG Level 2 pathway abundances. X‐axis: Abundance (KOs per million). Y‐axis: KEGG Level 2 pathways. The rightmost column indicates KEGG Level 1 categories. Values represent mean abundance across all samples. (D) Differential KEGG metabolic pathways (intergroup comparison). X‐axis: log_2_(fold change) (positive = upregulation in cancer; negative = downregulation). Y‐axis: KEGG pathways. Color intensity denotes statistical significance. (E) Taxon‐resolved MetaCyc pathway contributions. X‐axis: Samples (grouped/ordered by compositional similarity). Y‐axis: Relative pathway abundance. Stacked bars show genus‐level contributions of taxa to each metabolic pathway.

Building upon the identified structural disparities in the microbiota, functional prediction was performed for keystone taxa to elucidate their metabolic potential and mechanisms of host interaction. Consistent with the foregoing taxonomic analyses, Lactobacillus exhibited a widespread negative association with Prevotella, reflecting competitive exclusion between a health‐associated commensal and potential pathogens. Within the cancer cohort, multiple Prevotella nodes were positively interconnected, forming a cooperative pathobiont module. Healthy samples (group C) were centered on a Lactobacillus‐driven stable network, whereas cervical‐cancer samples (group A) displayed a Prevotella‐centered pathogenic network of greater complexity, highlighting Prevotella as a keystone genus in cervical carcinogenesis (Figure 3A ).

The rewired interaction landscape confirmed a fundamental restructuring of the microbiome. To translate these topological shifts into functional consequences, principal coordinate analysis (PCoA) was conducted on Bray–Curtis distances computed from enzyme commission and KEGG orthologue (KO) profiles. PCoA revealed a pronounced functional separation between groups, corroborating disease status as the primary driver of functional divergence. Functional dispersion within each group was evident, potentially reflecting inter‐individual variability among patients (Figure 3B). PICRUSt2 was subsequently applied to predict the relative abundances of KEGG level‐2 pathways. Metabolism dominated both cohorts, with carbohydrate, energy, and lipid metabolism representing the core metabolic backbone necessary for microbial proliferation and host–microbe crosstalk. Carbohydrate metabolism: glycolytic flux was significantly elevated in cervical‐cancer samples, supporting tumor‐microenvironment acidification (lactate accumulation) and potentially promoting immune evasion and cancer‐cell invasion. Energy metabolism: oxidative phosphorylation was diminished in the cancer cohort, indicating a metabolic shift toward anaerobic fermentation.

Immune‐system pathways: although low in abundance, antigen‐presentation and complement cascades were detected. Their reduced representation may reflect microbial strategies of immune evasion (Figure 3C). Among the differential KEGG pathways, the dominant module was “Tropane, piperidine and pyridine alkaloid biosynthesis,” a secondary‐metabolism route whose products can exert anti‐inflammatory, pro‐tumorigenic, or neurotoxic effects. Within this module, the sub‐pathway “Dioxin degradation” (microbial detoxification of dioxins) was significantly down‐regulated in cervical‐cancer samples, suggesting diminished clearance of environmental toxins and heightened host exposure (Figure 3D).

To identify the microbial executors underlying each differential pathway, MetaCyc annotations were integrated to construct taxon‐resolved pathway contribution maps. The AEROBACTINSYN‐PWY pathway was markedly up‐regulated in the cancer cohort (Figure 3E).

## 4 Discussion

### 4.1 Summary of Main Results

Leveraging 16S rRNA sequencing, this study systematically compared the structure, composition, and function of the reproductive tract microbiota between cervical cancer patients and healthy controls in Southern China. The cancer group exhibited significantly elevated α/β‐diversity, reduced Lactobacillus, enriched Prevotella, and functional profiles consistent with activated glycolysis and immunosuppressive pathways (Hu et al. 2024; Wang et al. 2024; Zhang et al. 2021). These findings confirm microbiota dysbiosis as a key cofactor in cervical carcinogenesis, providing new insights into its etiology and potential microecological interventions. While all cervical cancer cases were positive for high‐risk HPV, the healthy controls were universally HPV‐negative. By capitalizing on this clear contrast, our study design enabled a precise delineation of the cervical microbial alterations specifically attributable to HPV as the primary etiological factor. Consequently, we posit that a persistent, carcinogenic high‐risk HPV infection creates a selective pressure, which is beneficial to the growth of bad bacteria, characterized by the depletion of *Lactobacillus* and enrichment of anaerobic taxa such as *Prevotella*, as observed in our results. No significant differences in microbial community structure were observed between the different HPV subtype infection groups (HPV16+ group vs. HPV + /non‐16 group). However, both differed significantly from the HPV‐ group. This suggests that the impact of high‐risk HPV infection on the cervical microenvironment, regardless of subtype, may outweigh the effects of inter‐subtype variability, and common pathways in HPV‐induced carcinogenesis may be associated with microbial dysbiosis. Our findings provide critical targets and a foundational framework for developing microbial biomarkers as auxiliary diagnostic or prognostic tools.

We compared our results with data from other geographical regions. The observed increase in alpha diversity, depletion of *Lactobacillus*, and enrichment of *Prevotella* in cervical cancer patients in our study are consistent with the global meta‐analysis results by Peng et al. (2025), which confirms that the abundance of *Prevotella* is significantly higher in cervical cancer patients than in HPV‐negative individuals (Peng et al. 2025). When our results are compared with those from other Chinese cohorts, both similarities and differences are observed. Consistent with our results, Li et al. (2023) have reported enrichment of *Prevotella*, *Gardnerella*, and *Sneathia* in cervical cancer patients from Southwest China (Li et al. 2023). However, the relative abundance of specific taxa is different across various regions. For instance, Cheng et al. (2024) have found that clinical factors such as pH > 5 and H_2_O_2_ levels are strongly associated with microbial beta‐diversity in a Northern Chinese cohort, highlighting the interaction between the local microenvironment and microbial composition (Cheng et al. 2024). Our functional predictions, including activated glycolysis and immunosuppressive pathways, align with multi‐omics findings from both Chinese and international cohorts (Han et al. 2024; Yang et al. 2025). Furthermore, comparisons with non‐Asian populations revealed more significant differences. Łaniewski et al. (2024) have studied native American women and found that only 44% exhibited the dominance of *Lactobacillus* (compared to 58% in non‐native women), and observed associations between sociodemographic factors (multiple household members, lower education, and high parity) and vaginal dysbiosis (Łaniewski et al. 2024). These findings suggest that microbial patterns associated with cervical cancer are not uniform across populations. These geographical differences may be attributable to host genetic background (genetic polymorphisms affecting immune responses and microbial colonization), differences in HPV subtype distribution, sociodemographic and behavioral factors (high parity, hygiene practices, contraceptive methods), environmental factors (high‐sugar or high‐fat diets influencing systemic metabolism and local microbiota), and methodological factors (sampling site, choice of 16S rRNA hypervariable region, and control for variables such as HPV subtype, CIN (cervical intraepithelial neoplasia) stage, and antibiotic use history).

### 4.2 Results in the Context of Published Literature

The development of cervical cancer is strongly linked to HPV infection (Zhang et al. 2024). However, only a minority of HPV‐infected individuals progress to malignancy (P. Wang et al. 2024), implicating that cofactors such as microbiota in the reproductive tract may play a role. Previous studies have established a relationship between CIN severity and vaginal microbial dysbiosis. In a large cohort study, Mitra et al. (2015) have demonstrated that increased CIN grade was associated with higher alpha diversity and progressive depletion of *Lactobacillu* (Mitra et al. 2015). Similarly, a systematic review by Peng et al. (2025) has observed that the abundance of *Prevotella* is significantly higher in cervical cancer patients than in HPV‐negative individuals (Peng et al. 2025). Consistent with these findings, our results show that cervical cancer patients exhibited significantly higher alpha diversity, marked depletion of *Lactobacillus* (from 72.5% to 12.5%), and enrichment of *Prevotella* (from 6.8% to 28.3%) compared to healthy controls. This dysbiosis is thought to promote HPV persistence through chronic inflammation. Mechanism studies have demonstrated the association between dysbiosis‐associated bacteria (e.g., Prevotella, Gardnerella) and the activation of the TLR4/IL‐8 pathway, which in turn drives the production of pro‐inflammatory cytokines and impairs antiviral immunity (Agarwal et al. 2015; Dong et al. 2023; Sekaran et al. 2023). Our functional predictions support this mechanism, revealing the upregulation of TLR4/NF‐κB pathway in the cancer group, which is consistent with prior reports linking dysbiosis to pro‐inflammatory signaling. Moreover, enrichment of siderophore biosynthesis (e.g., AEROBACTINSYN‐PWY) in our cohort suggests a novel link between dysbiosis, iron acquisition, and tumor progression. This finding is consistent with the viewpoint that iron metabolism is intimately linked to tumor biology and immune regulation (Jiang et al. 2023; Pfeifhofer‐Obermair et al. 2018). Our data directly corroborate recent findings. Bautista et al. (2025) have reported that community state type (CST) IV (low *Lactobacillus*, high anaerobes) predicts persistent HR‐HPV and lesion progression (Bautista et al. 2025). The non‐*Lactobacillus*‐dominated profile with elevated *Prevotella*, *Gardnerella*, and *Sneathia* in our cancer group is similar to CST IV, further supporting the role of specific microbial communities in carcinogenesis related to HPV.

### 4.3 Strengths and Weaknesses

The novelty of our work is multifaceted. First, we investigate a Southern Chinese cohort, a population underrepresented in prior (largely Eurocentric) studies, thereby providing critical region‐specific data and expanding the geographical scope of cervicovaginal microbiome research in oncology. Second, through a systematic comparison of the cervical and vaginal microbiota within this homogeneous cohort, we demonstrate that cancer‐associated dysbiosis presents consistent features across anatomical niches. Third, by applying a multi‐dimensional, integrated analysis to our 16S rRNA sequencing data, we elucidate not just the structural imbalance but also the key bacterial taxa implicated in cervical cancer, offering a perspective beyond alpha‐ and beta‐diversity metrics. Finally, by incorporating functional prediction, we bridge the observed shifts in microbial community metabolism with the remodeling of the cervical immune microenvironment, proposing a tangible link between microbiome function and carcinogenesis. However, several limitations should be noted. First, the study excluded precancerous cases (including cervical intraepithelial neoplasia, abbreviated as CIN), thus precluding elucidation of microbiota dynamics during cancer progression. Second, 16S rRNA sequencing inherently lacks resolution for species‐level functional heterogeneity, necessitating metagenomic validation. Third, the cross‐sectional design precludes causal inference, highlighting the need for prospective cohorts to validate vaginal microbiota as diagnostic or predictive biomarkers. The lack of comparative data from other Chinese regions or global populations precludes definitive conclusions on the geographic specificity of these microbial patterns. Therefore, rather than asserting regional uniqueness, our findings emphasize a prudent translational principle: candidate microbial biomarkers require validation within the specific demographic context intended for clinical use.

### 4.4 Implications for Practice and Future Research

Our findings indicated the depletion of *Lactobacillus*, enrichment of *Prevotella*, upregulation of siderophore biosynthesis (AEROBACTINSYN‐PWY), and the activation of the TLR4/NF‐κB pathway in the cancer group. In the future, metagenomics should be used to confirm aerobactin genes in *Prevotella* and other enriched taxa (Peng et al. 2025). Further research should establish HPV‐transgenic mouse models colonized with patient‐derived *Prevotella* strains to assess their role in tumor promotion via iron acquisition (le Roy et al. 1992). Additionally, it is necessary to perform metabolomic analysis of cervicovaginal fluids to quantify lactate and SCFAs, correlating with *Lactobacillus* abundance. It is also imperative to conduct in vitro co‐cultures of cervical epithelial cells with *Lactobacillus* versus *Prevotella* to assess barrier function and inflammatory responses. Furthermore, probiotic trials with *Lactobacillus* strains (e.g., L. crispatus) have been shown to inhibit *Prevotella* and restore acidic pH (Deschamps et al. 2025). Metabolic interventions targeting siderophore‐mediated iron acquisition (e.g., iron chelation, siderophore inhibitors) may be used as adjunctive therapy (le Roy et al. 1992). Moreover, multicenter longitudinal studies are needed to track microbiota dynamics, HPV persistence, and lesion progression in diverse cohorts to establish causality (Tsementzi et al. 2025). Future research should integrate host genotyping to identify genetic modifiers of the relationship between microbiota and cancers.

## Author Contributions

**Guojiao Wang:** writing – original draft preparation, writing ‐ review and editing, conceptualization, methodology; **Huadong Tian:** conceptualization, writing – review and editing; **Yan Li:** methodology, writing – review and editing; **Zimo Wang:** formal analysis and investigation, writing – review and editing; **Xiaotong Liu:** formal analysis and investigation, writing – review and editing; **Yuexi Luo:** funding acquisition, resources, supervision, writing – review and editing.

## Ethics Statement

The study was performed following the principles outlined in the Declaration of Helsinki and obtained approval from the Ethics Committee of Nanchong Hospital, Capital Medical University & Beijing Anzhen Hospital (approval no. 2024‐Review‐030). Written informed consent was obtained from all participants.

## Conflicts of Interest

The authors declare no conflicts of interest.

## Data Availability Statement

16S rRNA sequencing data for all the samples have been deposited in NCBI with the accession number of PRJNA1433670.

## Supporting information


**Figure S1:** Taxonomic Tree with Abundance Information.
**Figure S2:** PCoA Plot: Two‐Dimensional Ordination of Samples.
**Table S1:** Demographic and clinical characteristics of participants.
