# Supplementary material for: Exploring Cervical Cancer‐Associated Vaginal and Cervical Microbiota via 16S rRNA Sequencing
Source: Microbiologyopen. 2026 Sep 21;15(5):e70412. doi: 10.1002/mbo3.70412 (PMC13592373; doi:10.1002/mbo3.70412)
Supplement: Supplementary file 1 [file MBO3-15-e70412-s001.docx]

**Table S1** Demographic and clinical characteristics of participants

|  | **Cancer** | **Control** | **P-value** |
| --- | --- | --- | --- |
|  | **N=16** | **N=16** |  |
| Age(years) | 54.2 (7.79) | 53.2 (3.83) | 0.629^a^ |
| BMI**(kg/m²)^d^** | 24.6 (4.28) | 24.1 (3.68) | 0.727^a^ |
| Smoke: N | 16 (100%) | 16 (100%) | -^b^ |
| Drink: N | 16 (100%) | 16 (100%) | -^b^ |
| Number of pregnancies | 3.31 (1.62) | 3.44 (1.21) | 0.807^a^ |
| Number of deliveries | 2.19 (1.11) | 1.38 (0.72) | 0.021^a^ |
| Number of abortions | 1.12 (1.50) | 2.06 (1.34) | 0.072^a^ |
| Age at menarche(years) | 13.6 (1.02) | 13.3 (1.01) | 0.393^a^ |
| Menstrual cycle frequency^c^ (days) | 28.8 (2.59) | 29.4 (3.34) | 0.599^a^ |
| Menstrual duration (days) | 6.06 (1.18) | 5.50 (0.97) | 0.151^a^ |

Data are presented as Mean (SD) for continuous variables and n (%) for categorical variable.

^a^ P-values were calculated using Student's t-test for continuous variables and the chi-square test was applied for categorical variables

^b^ Statistical testing was not applicable as the characteristic was invariant across groups (all participants reported no smoking/alcohol consumption).

^c^ "Frequency" refers to the average length of the menstrual cycle in days.

^d^ BMI: body mass index

**Figure S1** Taxonomic Tree with Abundance Information

The pie charts at the branch nodes of the taxonomic tree (threshold: 0.5%) display the composition proportion of each taxonomic unit across samples or groups. A larger sector area indicates a higher relative abundance of the taxonomic unit in the corresponding sample or group.

**Figure S2** PCoA Plot: Two-Dimensional Ordination of Samples

Each point in the plot represents an individual sample, with different colors indicating distinct sample groups. The percentages in parentheses on the axes represent the proportion of variance in the sample dissimilarity (distance matrix) explained by the corresponding axis. The closer the two points are along an axis, the more similar the microbial community composition of the corresponding samples along that dimension.
